# Development and evaluation of artificial intelligence tools to estimate volumetric breast density from processed 2D mammograms

**DOI:** 10.1093/bjrai/ubag009

**Published:** 2026-04-28

**Authors:** Sam Ellis, Sandra Gomes, Mark D Halling-Brown, Kenneth C Young, Lucy M Warren

**Affiliations:** Department of Scientific Computing, Royal Surrey NHS Foundation Trust, Guildford GU2 7XX, United Kingdom; Department of Scientific Computing, Royal Surrey NHS Foundation Trust, Guildford GU2 7XX, United Kingdom; Department of Scientific Computing, Royal Surrey NHS Foundation Trust, Guildford GU2 7XX, United Kingdom; Centre for Vision, Speech and Signal Processing, University of Surrey, Guildford GU2 7XH, United Kingdom; National Co-ordinating Centre for the Physics of Mammography, Royal Surrey NHS Foundation Trust, Guildford GU2 7XX, United Kingdom; Department of Physics, University of Surrey, Guildford GU2 7XH, United Kingdom; Department of Scientific Computing, Royal Surrey NHS Foundation Trust, Guildford GU2 7XX, United Kingdom

**Keywords:** breast density estimation, artificial intelligence, mammography

## Abstract

**Objectives:**

Artificial intelligence (AI) has shown promise for estimating volumetric breast density values from processed, “for presentation,” mammograms. However, previous evaluations have typically used small datasets or focused on a single vendor. In this study, we aimed to improve volumetric breast density estimation from processed mammograms for the three main UK vendors with a combination of improved training methods and the utilization of up-to-date data from the large OPTIMAM Mammography Image Database (OMI-DB).

**Methods:**

Paired processed/unprocessed mammograms were obtained from OMI-DB. Ground-truth, image-level density values were calculated by passing unprocessed images through a commercial density estimation tool. AI tools, comprising feed-forward convolution neural networks, were then trained to reproduce these values from the corresponding processed mammograms.

**Results:**

Patient-level AI predictions for volumetric breast density demonstrated strong correlation with ground-truth values derived from unprocessed image counterparts (*r* = 0.954-0.976). Models trained on less prevalent manufacturers performed worse (*r* = 0.954 compared to 0.976 for the most prevalent manufacturer), highlighting the importance of collecting larger training datasets in future. Error levels were higher in patients with dense breasts. Model performance was generally consistent across screening sites but correlated with patient age, possibly due to the correlation of age and breast density.

**Conclusions:**

The presented models demonstrated good performance overall and were generally consistent across screening sites.

**Advances in knowledge:**

The presented AI tools provide a means of estimating breast density from processed mammograms, enabling further research into breast cancer epidemiology and risk where only processed mammograms are available.

## Introduction

In mammographic imaging, *breast density* refers to the amount or proportion of fibroglandular tissue present in the breast. These quantities are important for characterizing a woman’s risk of breast cancer.^1–4^ In general, an increased cancer risk from higher breast density can be attributed to an increased base risk of cancer development^5^ and a higher risk of existing cancers being “masked” by dense tissue, leading to missed detections during screening.^6^^,^^7^ These mechanisms manifest as differences in observed breast cancer risk when considering different density measures and cancer types.^5^^,^^8^^,^^9^

For these reasons, there is a growing trend to account for breast density in breast cancer risk models and screening protocols, either directly by including numeric values of density into models,^3^^,^^10^ or by altering screening protocols for women identified as having dense breasts.^11^ Particular focus has been given to the assessment of breast density from 2-dimensional mammograms (mammographic breast density) due to the ubiquity of mammographic imaging within screening programmes.^12^

There have been many approaches proposed for the characterization of mammographic breast density. Area-based approaches include reader-assessed density either via discrete categories (eg, BI-RADS density grades) or semi-continuous visual analog scales^2^^,^^13^; and automated continuous variants that broadly calculate the proportion of “bright” pixels in the breast compared to the total number of pixels in the breast.^14^^,^^15^ Alternatively, volumetric quantities, such as fibroglandular volume (FGV), breast volume (BV), and volumetric breast density (VBD, defined as the ratio of FGV to BV), can be estimated from raw mammographic projection data with the use of physics-based models of image acquisition coupled with additional quantities typically stored in image headers, such as breast thickness, exposure levels, and detector characteristics.^16^ These physics models specifically require unprocessed mammograms since the pixel values are proportional to incident x-ray flux, which gives information on the density of occluding tissue. By contrast, the proprietary and vendor-specific processing applied to render mammograms suitable for human reading includes various enhancements that degrade the physical information contained in a mammogram.

However, in many breast screening programmes, including the UK National Health Service (NHS) breast screening programme, unprocessed mammographic data is not routinely available. This can be for a variety of reasons, for example, insufficient infrastructure or technical expertise at screening sites. For this reason, volumetric measures such as VBD and FGV have proven more difficult to apply in practice, limiting their use in the literature. Nonetheless, volumetric measures are still of interest, and perhaps even preferable when studying the epidemiology of breast cancer, due to the possibility of volume effects being particularly related to breast cancer risk.^5^

To this end, previous work has attempted to use artificial intelligence (AI) to create models capable of estimating FGV and VBD directly from processed mammograms.^17–19^ These models allow for retrospective epidemiological studies of the relationship between FGV and VBD with breast cancer, where only processed mammograms are available.

This work improves on the previously described AI tools for FGV and VBD estimation from processed mammograms^18^ and provides detailed evidence of their accuracy and potential suitability for use in future research and clinical studies. The specific contributions of this work are the following:

1. Leveraging the most up-to-date and more extensive data available from the OPTIMAM mammography image database^20^ to train models using large numbers of mammograms not previously available.
2. Improving AI training by exploiting spatial information contained within images and headers to improve performance.
3. Development and evaluation of bespoke AI tools for each of the three major vendors of mammographic equipment in the OPTIMAM image database: Hologic, Siemens, and GE.

This article is structured as follows: The “Methods” section details the methodological aspects of the model development, such as data selection, curation, and splitting; model architecture and training procedure; and, finally, the metrics and techniques used to evaluate the produced models. The “Results” section presents the results showing model performance, including demographic breakdowns to contextualize performance values. The “Discussion” section discusses the results and highlights potential weaknesses of the models before finally presenting conclusions and suggesting areas for future work.

## Methods

### Dataset curation

In order to produce AI tools capable of estimating FGV and VBD directly from processed mammograms, we first searched the OPTIMAM image database for screening studies with paired unprocessed and processed data available. Unprocessed images were then passed through version 1.5.5 of the Volpara software (Volpara, Wellington, NZ), which uses a physics-based model^21^^,^^22^ to produce image-level ground-truth BV, FGV, and VBD values. These ground-truth values were then associated with the processed counterparts of the mammograms, and the unprocessed images were subsequently discarded. The paired processed images/volumetric measures were then used for model training and evaluation. Images that Volpara failed to process were excluded from the study.

From the available images for which ground-truth information was available, three datasets were curated, one for each manufacturer, each containing no more than one study per patient. Since the image processing significantly affects image appearance, the Hologic and Siemens datasets were approximately balanced by image processing software version. As there was much less available GE data and there was a smaller number of GE software versions, the GE dataset was not balanced by software version. Finally, training, validation, and test datasets were produced per manufacturer by splitting the data in a 60:20:20 ratio at the patient level. Note that a single patient could be included in the datasets for multiple manufacturers if they had studies from different manufacturers.

### Model training

FGV and VBD are both of interest for quantifying breast cancer epidemiology and risk. In order to produce consistent estimates of FGV and VBD, AI models were trained in pairs: one model to produce estimates of BV and the other to produce estimates of FGV. From the outputs of these models, VBD is estimated as FGV/BV. Furthermore, since different mammographic views have very different appearances due to the inclusion of the pectoral muscle, separate models were trained for the 2 most common views: cranial-caudal (CC) and medial-lateral oblique (MLO). This resulted in 4 AI models per manufacturer, denoted BV-CC, BV-MLO, FGV-CC, and FGV-MLO.

The same AI architecture was used for training all models, based on an architecture previously shown to be effective for volumetric density estimation^18^ (Appendix SI). Individual 2D mammograms are passed through a convolutional neural network backbone, which outputs a vector representation for each image. This vector is then passed through a series of fully connected layers along with the compressed breast thickness as recorded in the DICOM header to produce a single scalar value per image. We note that including the compressed breast thickness as a model input is a novel innovation not previously reported in the literature.

The models were trained using mean-squared-error (MSE) loss with a batch size of 2, optimized with Adam. Suitable learning rates were determined using a learning-rate finder and set to 1E−4 for all BV models, 7E−5 for all FGV-CC models, and 6E−5 for all FGV-MLO models. Before being passed to the AI model, images were rescaled to a 0.09 mm isotropic pixel size and padded/cropped to achieve the required 3328 × 2560 grid size. Padding and cropping were performed opposite the breast (according to the laterality recorded in the DICOM header) so that the chest wall remained adjacent to one side of the image. For each training run, model inputs (ie, pixel intensity values and compressed breast thicknesses) were normalized to zero mean and unit standard deviation.

To reflect the varying amounts of data available for each manufacturer, Hologic models were trained for 15 epochs, Siemens models for 35, and GE models for 50. In all cases, validation performance was evaluated at the end of each training epoch by calculating the MSE between model outputs and ground-truth values for the corresponding validation dataset. The model from the epoch with the lowest validation MSE was selected and applied to the corresponding test set for further analysis.

### Model evaluation

Final model performance for BV, FGV, and VBD prediction was evaluated on the test datasets via a range of metrics, including MSE, Pearson’s correlation coefficient, and the gradient and intercept obtained from a linear regression of model outputs against ground-truth values. These performance metrics were also calculated at the patient level for each manufacturer by averaging target and predicted values across all available images for a patient. Analyses were also stratified by other factors of interest, such as patient age, screening site, and image processing software version. 95% confidence intervals for MSE and correlation coefficients were estimated as the 2.5th and 97.5th percentiles of bootstrap distributions calculated using 1000 resamplings.

## Results

### Dataset characteristics

Table 1 provides the final breakdown of the training, validation, and test datasets for each manufacturer by patient age, reported ethnic background, screening site, and study year. Variations were observed between the manufacturers, reflecting the status of the OPTIMAM image database at the time of data curation. For example, the majority of Siemens data was from 2023 and 2024, whereas GE data were older, with only very few studies dated later than 2019.

**Table 1:** Final training, validation, and test datasets, including breakdowns by age, screening site, ethnic background, and study year.

	Hologic			Siemens			GE		
	Training	Validation	Test	Training	Validation	Test	Training	Validation	Test
Overall	27 720	9246	9247	9641	3215	3217	4891	1631	1630
**Age, years**									
0-49	1014 (3.7)	353 (3.8)	333 (3.6)	79 (0.8)	27 (0.8)	22 (0.7)	240 (4.9)	78 (4.8)	82 (5.0)
50-55	7999 (28.9)	2644 (28.6)	2606 (28.2)	2709 (28.1)	918 (28.6)	864 (26.9)	1492 (30.5)	494 (30.3)	497 (30.5)
56-60	6537 (23.6)	2168 (23.4)	2142 (23.2)	2269 (23.5)	726 (22.6)	768 (23.9)	1044 (21.3)	336 (20.6)	347 (21.3)
61-65	5309 (19.2)	1767 (19.1)	1861 (20.1)	2112 (21.9)	711 (22.1)	723 (22.5)	920 (18.8)	326 (20.0)	302 (18.5)
66-70	4812 (17.4)	1596 (17.3)	1597 (17.3)	1861 (19.3)	615 (19.1)	654 (20.3)	790 (16.2)	275 (16.9)	274 (16.8)
71+	2047 (7.4)	717 (7.8)	708 (7.7)	611 (6.3)	218 (6.8)	186 (5.8)	392 (8.0)	119 (7.3)	124 (7.6)
N/A	2 (0.0)	1 (0.0)	0 (0.0)	0 (0.0)	0 (0.0)	0 (0.0)	13 (0.3)	3 (0.2)	4 (0.2)
**Site**									
Site 1	15 283 (55.1)	5185 (56.1)	5167 (55.9)	0 (0.0)	0 (0.0)	0 (0.0)	3044 (62.2)	1006 (61.7)	1042 (63.9)
Site 2	2176 (7.8)	715 (7.7)	671 (7.3)	0 (0.0)	0 (0.0)	0 (0.0)	15 (0.3)	4 (0.2)	4 (0.2)
Site 3	10 258 (37.0)	3345 (36.2)	3409 (36.9)	4383 (45.5)	1473 (45.8)	1434 (44.6)	1745 (35.7)	586 (35.9)	557 (34.2)
Site 4	0 (0.0)	0 (0.0)	0 (0.0)	5253 (54.5)	1742 (54.2)	1783 (55.4)	0 (0.0)	0 (0.0)	0 (0.0)
Other	3 (0.0)	1 (0.0)	0 (0.0)	5 (0.1)	0 (0.0)	0 (0.0)	87 (1.8)	35 (2.1)	27 (1.7)
**Ethnic background**									
Asian or Asian British	1716 (6.2)	590 (6.4)	574 (6.2)	275 (2.9)	96 (3.0)	88 (2.7)	224 (4.6)	72 (4.4)	75 (4.6)
Black or Black British	1277 (4.6)	450 (4.9)	415 (4.5)	178 (1.8)	45 (1.4)	54 (1.7)	32 (0.7)	12 (0.7)	14 (0.9)
Mixed	343 (1.2)	110 (1.2)	117 (1.3)	78 (0.8)	33 (1.0)	21 (0.7)	27 (0.6)	13 (0.8)	10 (0.6)
Unreported	2359 (8.5)	760 (8.2)	789 (8.5)	5539 (57.5)	1824 (56.7)	1883 (58.5)	628 (12.8)	218 (13.4)	184 (11.3)
Other Ethnic Groups	626 (2.3)	205 (2.2)	207 (2.2)	129 (1.3)	49 (1.5)	48 (1.5)	152 (3.1)	45 (2.8)	49 (3.0)
White	21 399 (77.2)	7131 (77.1)	7145 (77.3)	3442 (35.7)	1168 (36.3)	1123 (34.9)	3828 (78.3)	1271 (77.9)	1298 (79.6)
**Study year**									
2010	0 (0.0)	0 (0.0)	1 (0.0)	21 (0.2)	4 (0.1)	7 (0.2)	0 (0.0)	1 (0.1)	2 (0.1)
2011	62 (0.2)	12 (0.1)	16 (0.2)	1 (0.0)	1 (0.0)	0 (0.0)	51 (1.0)	19 (1.2)	12 (0.7)
2012	356 (1.3)	105 (1.1)	108 (1.2)	52 (0.5)	17 (0.5)	26 (0.8)	186 (3.8)	53 (3.2)	52 (3.2)
2013	1791 (6.5)	554 (6.0)	608 (6.6)	49 (0.5)	22 (0.7)	23 (0.7)	486 (9.9)	162 (9.9)	172 (10.6)
2014	3346 (12.1)	1164 (12.6)	1121 (12.1)	1 (0.0)	0 (0.0)	0 (0.0)	2221 (45.4)	730 (44.8)	726 (44.5)
2015	1309 (4.7)	432 (4.7)	444 (4.8)	0 (0.0)	1 (0.0)	0 (0.0)	273 (5.6)	95 (5.8)	118 (7.2)
2016	4410 (15.9)	1474 (15.9)	1460 (15.8)	51 (0.5)	18 (0.6)	22 (0.7)	754 (15.4)	248 (15.2)	247 (15.2)
2017	4317 (15.6)	1447 (15.7)	1443 (15.6)	895 (9.3)	289 (9.0)	261 (8.1)	346 (7.1)	122 (7.5)	113 (6.9)
2018	3344 (12.1)	1101 (11.9)	1101 (11.9)	1344 (13.9)	447 (13.9)	414 (12.9)	244 (5.0)	80 (4.9)	84 (5.2)
2019	5604 (20.2)	1869 (20.2)	1862 (20.1)	1057 (11.0)	371 (11.5)	391 (12.2)	319 (6.5)	117 (7.2)	101 (6.2)
2020	158 (0.6)	64 (0.7)	51 (0.6)	28 (0.3)	15 (0.5)	13 (0.4)	8 (0.2)	4 (0.2)	3 (0.2)
2021	1607 (5.8)	566 (6.1)	566 (6.1)	657 (6.8)	211 (6.6)	225 (7.0)	3 (0.1)	0 (0.0)	0 (0.0)
2022	297 (1.1)	120 (1.3)	102 (1.1)	75 (0.8)	39 (1.2)	23 (0.7)	0 (0.0)	0 (0.0)	0 (0.0)
2023	1119 (4.0)	337 (3.6)	364 (3.9)	1135 (11.8)	345 (10.7)	352 (10.9)	0 (0.0)	0 (0.0)	0 (0.0)
2024	0 (0.0)	1 (0.0)	0 (0.0)	4275 (44.3)	1435 (44.6)	1460 (45.4)	0 (0.0)	0 (0.0)	0 (0.0)

Note that ethnicity information was retrieved from patients’ National Breast Screening System records, using the standard categories used throughout the NHS.^26^ Percentages are provided in parentheses.

### Overall AI test-set performance

**Figure 1:**
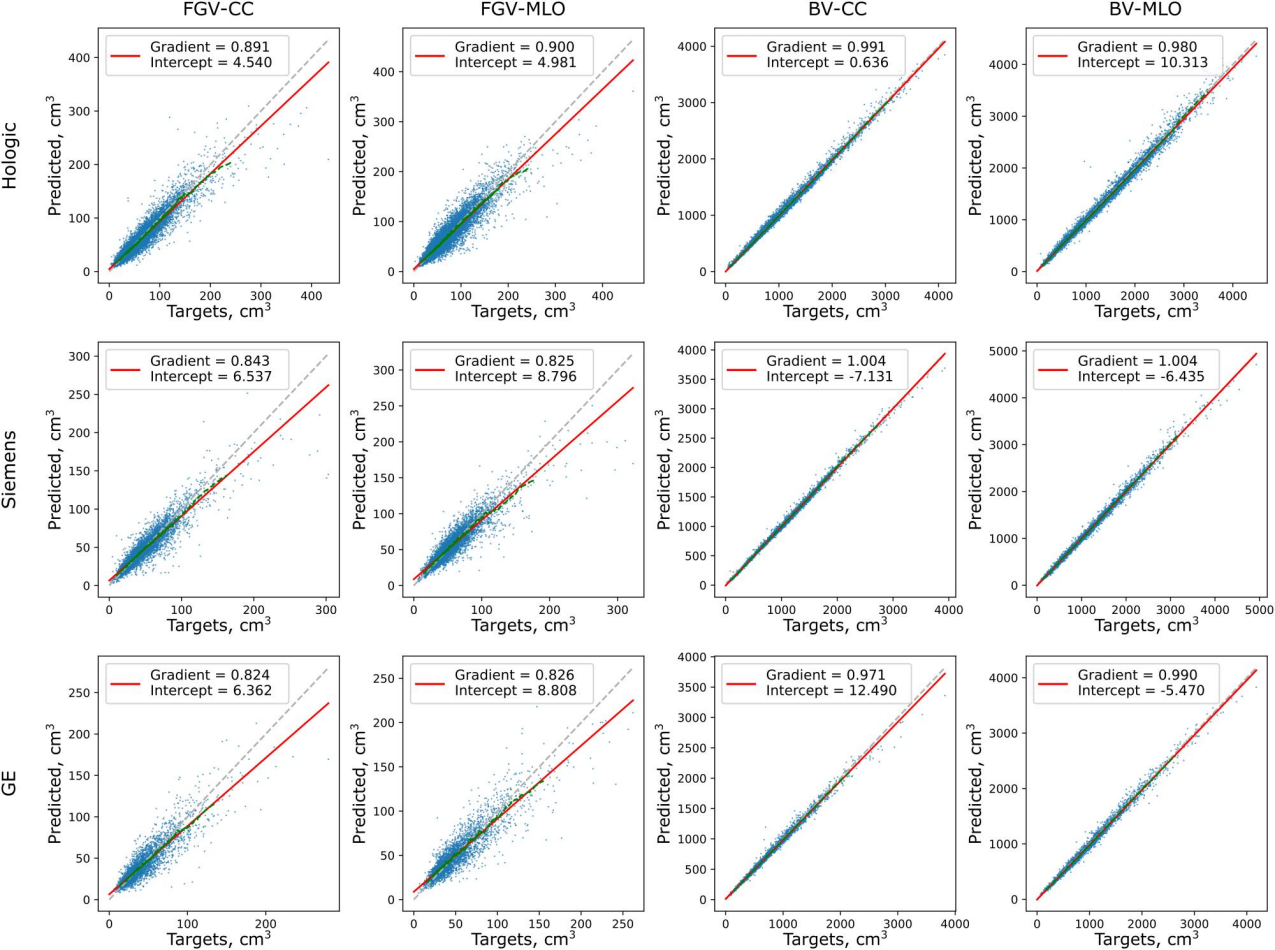
Scatter plots and line fits for each of the 12 AI models trained in this work. For each manufacturer, results are shown for FGV-CC, FGV-MLO, BV-CC, and BV-MLO. Lines of best fit are plotted in red, with gradient and intercept values shown. Dashed green lines show a moving average calculated every 100 samples.

Figure 1 shows the hold-out test-set performance of each of the 12 individual AI models selected based on the minimum per-epoch validation MSE (see Appendix SII for epoch-selection curves). Also shown are line fits in red and moving average curves in dashed green, generated by averaging every 100 points in the scatter plot. Corresponding MSE and correlation coefficients with 95% CIs are provided in Table 2. Generally, Hologic models trained using more data performed better than GE models, which had less data available. In addition, BV models consistently performed better than FGV models, which is to be expected given the particularly challenging nature of FGV estimation.

**Table 2:** MSE and correlation coefficient values for the models displayed in Figure 1, including 95% CIs calculated via bootstrapping.

		MSE	Correlation
**Hologic**	BV-CC	982 (934-1033)	0.998 (0.998-0.998)
	BV-MLO	2453 (2263-2695)	0.997 (0.997-0.997)
	FGV-CC	106 (97-115)	0.947 (0.943-0.950)
	FGV-MLO	148 (139-157)	0.934 (0.930-0.937)
**Siemens**	BV-CC	752 (688-825)	0.999 (0.999-0.999)
	BV-MLO	2178 (1972-2395)	0.997 (0.997-0.998)
	FGV-CC	103 (90-118)	0.925 (0.917-0.933)
	FGV-MLO	126 (113-142)	0.922 (0.914-0.928)
**GE**	BV-CC	1865 (1597-2174)	0.996 (0.995-0.996)
	BV-MLO	2853 (2588-3130)	0.996 (0.995-0.996)
	FGV-CC	144 (130-160)	0.892 (0.880-0.903)
	FGV-MLO	169 (151-188)	0.899 (0.889-0.910)

**Figure 2:**
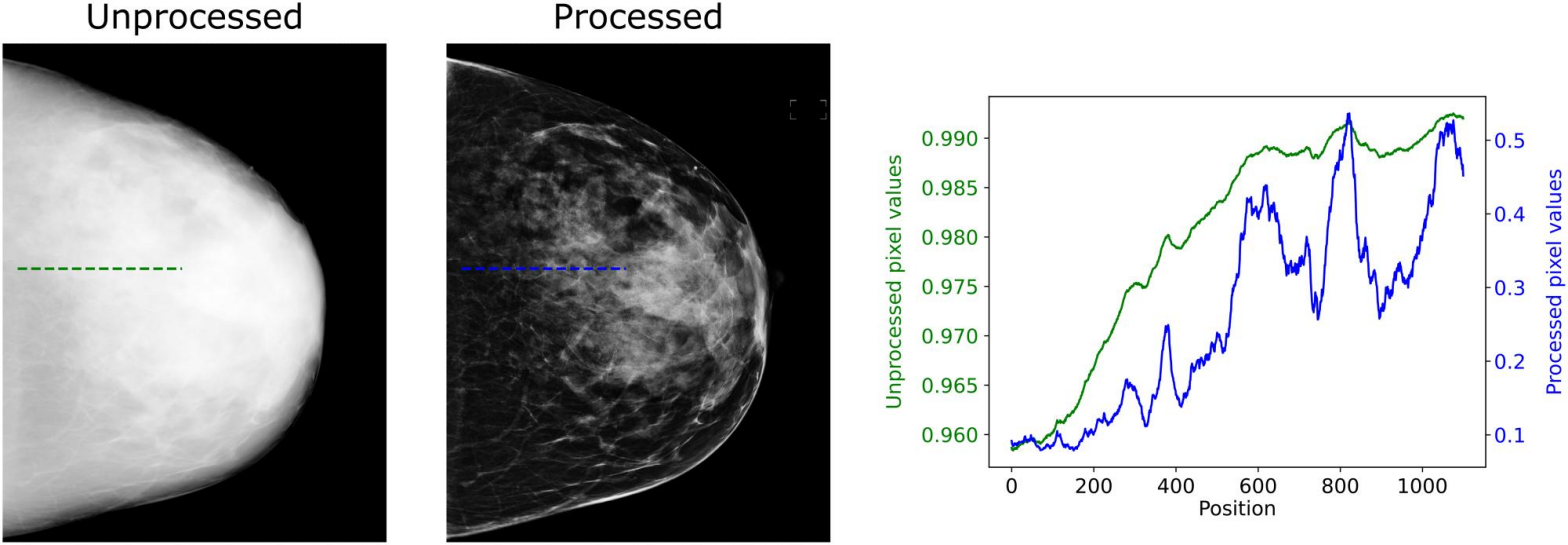
High-FGV Hologic example test case, showing both the original unprocessed from which the ground-truth FGV was derived (left) and the processed mammogram provided to the AI model (middle). Line profiles (right) show the pixel values along a line progressing from non-dense to dense tissue. The image processing boosts local contrast for presentation to readers, causing a greater contrast in dense regions. This effect may contribute to the tendency of AI models to underestimate FGV at higher ground-truth values (Figure 1). For this image, the ground-truth FGV was 433 cm^3^, and the AI predicted a value of 209 cm^3^. *Note*: the unprocessed image was windowed [80%, 100%] of original intensity values for visualization.

We observed that for all FGV models, there was a tendency to underestimate FGV at higher ground-truth values. Visual inspection of images in this regime alongside their unprocessed counterparts (Figure 2) showed that for these large, dense breasts the image processing appeared to enhance local contrast, making dense pixels appear less dense, possibly contributing to the underestimation of FGV from the AI models.

**Figure 3:**
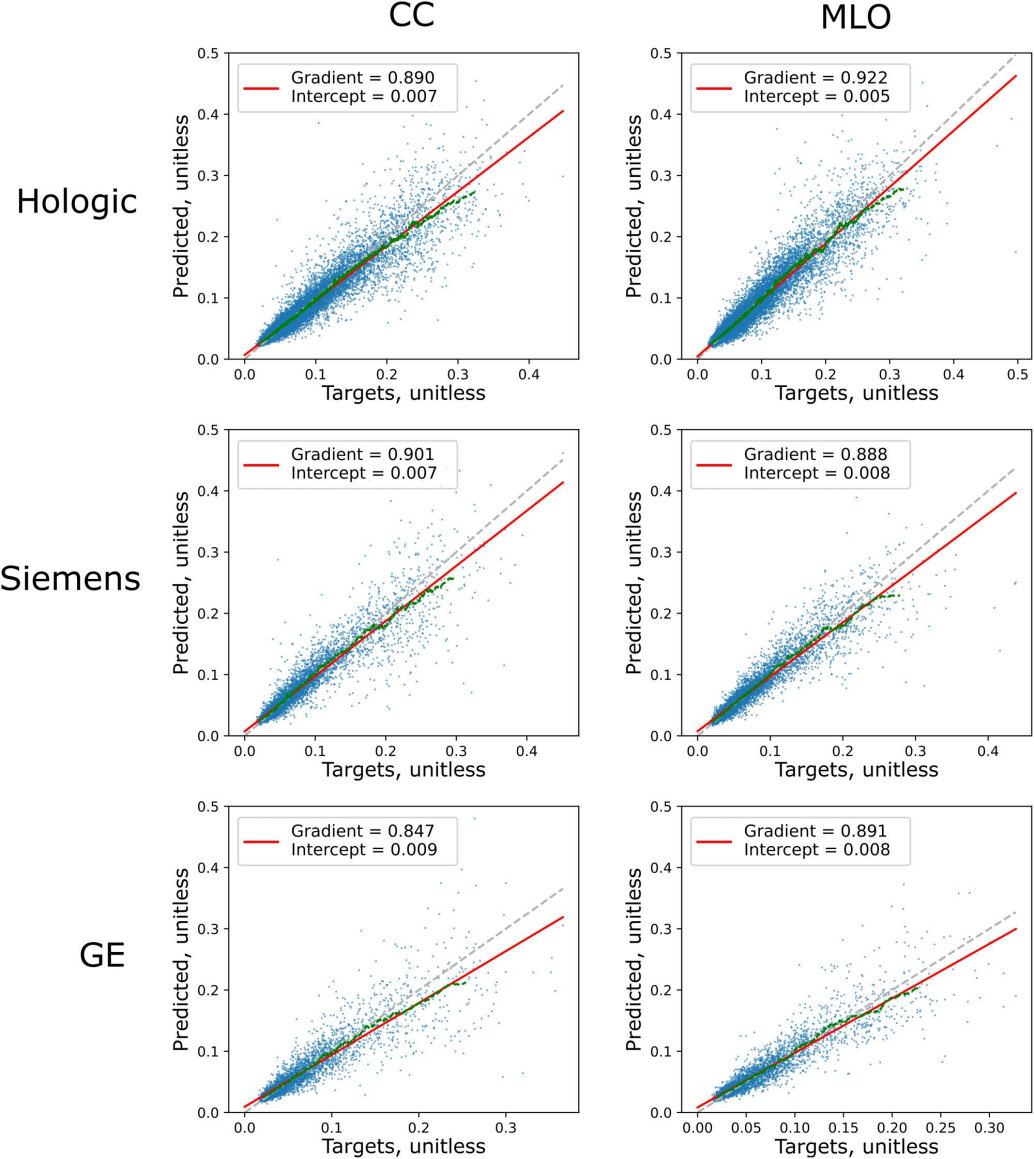
Scatter plots and line fits for VBD. Image-level VBD predictions were produced by calculating the ratio of FGV to BV for each manufacturer-view combination. Red lines show lines of best fit, with the displayed gradient and intercepts, and green dashed lines show a moving average calculated every 100 samples.

When considering model performance in terms of VBD (Figure 3), similar trends were observed: poorer performance for manufacturers with less data and at higher VBD values.

**Figure 4:**
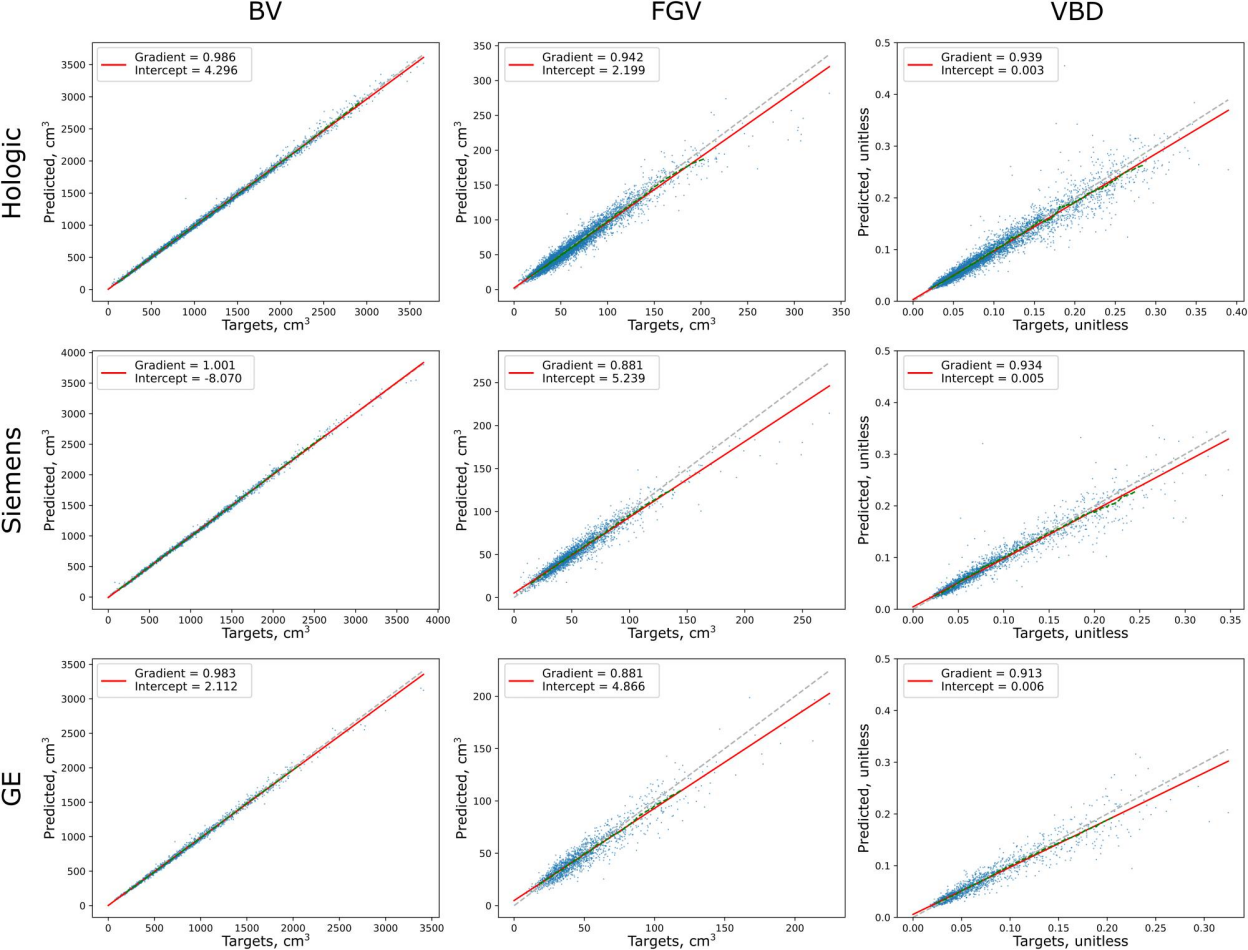
Patient-level BV, FGV, and VBD results calculated by averaging target and predicted values across all images available per patient.

Figure 4 shows the patient-level BV, FGV, and VBD results for each of the manufacturers, created by averaging values from both the CC and MLO models. Patient-level performance was generally superior to image-level performance due to the averaging process. Again, GE models performed worst due to the low amount of data available. Patient-level model output correlated strongly with ground-truth values, with similar correlation coefficients observed for both FGV and VBD estimation (Table 3). This is because BV models generally performed well so that the majority of the error in VBD estimation arises from errors in FGV values.

**Table 3:** Patient-level correlation coefficients, with 95% CIs, for the trained AI models.

	Patient-level correlation		
	BV	FGV	VBD
**Hologic**	0.999 (0.999-0.999)	0.976 (0.975-0.978)	0.974 (0.969-0.977)
**Siemens**	0.999 (0.999-0.999)	0.968 (0.964-0.972)	0.965 (0.956-0.973)
**GE**	0.998 (0.998-0.998)	0.954 (0.947-0.959)	0.954 (0.947-0.961)

### Performance breakdown

**Figure 5:**
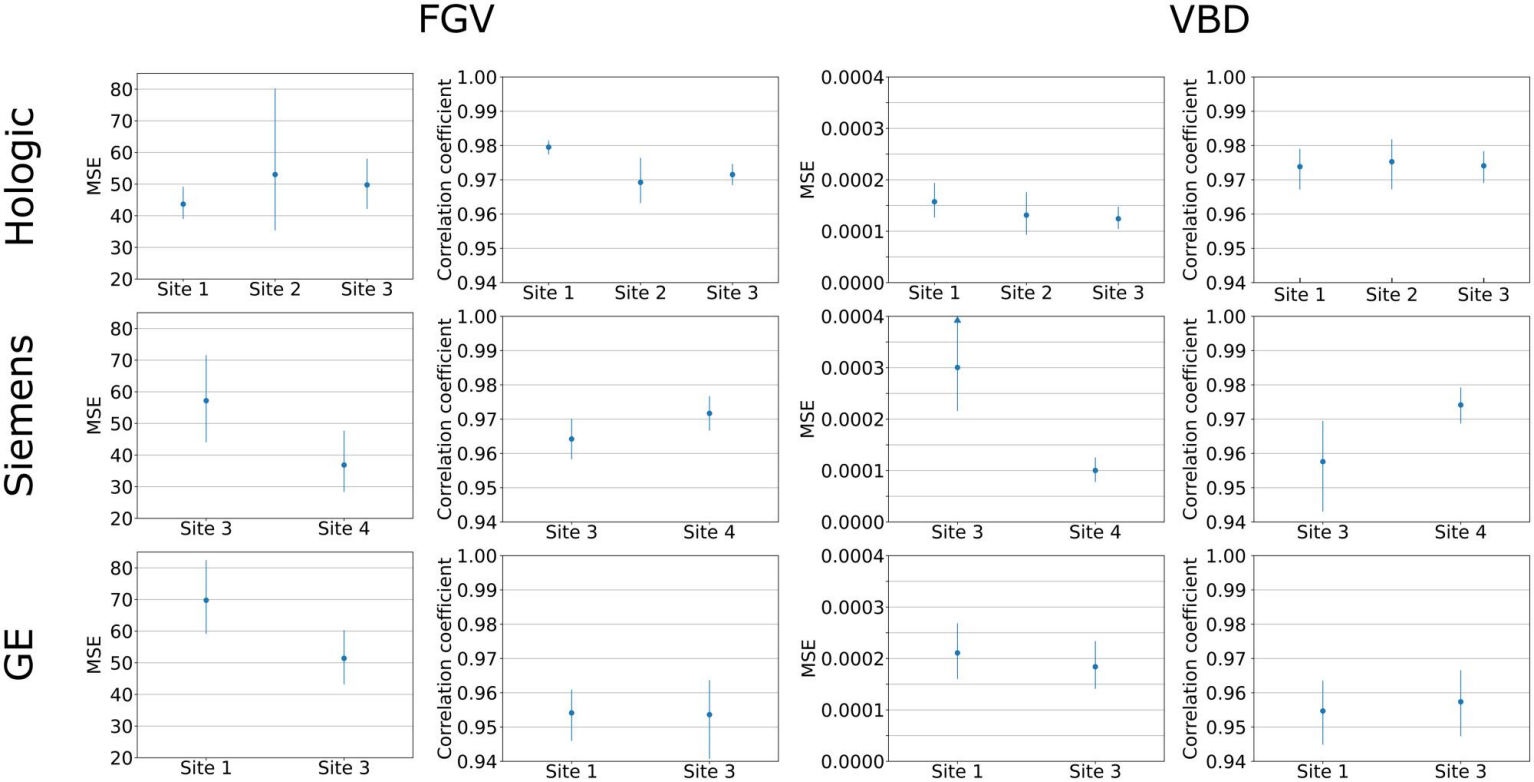
Patient-level FGV and VBD mean-squared-error (MSE) and correlation coefficients, stratified by screening site. Error bars show 95% CIs. Note that for each manufacturer sites with <50 test patients were excluded for clarity.

**Figure 6:**
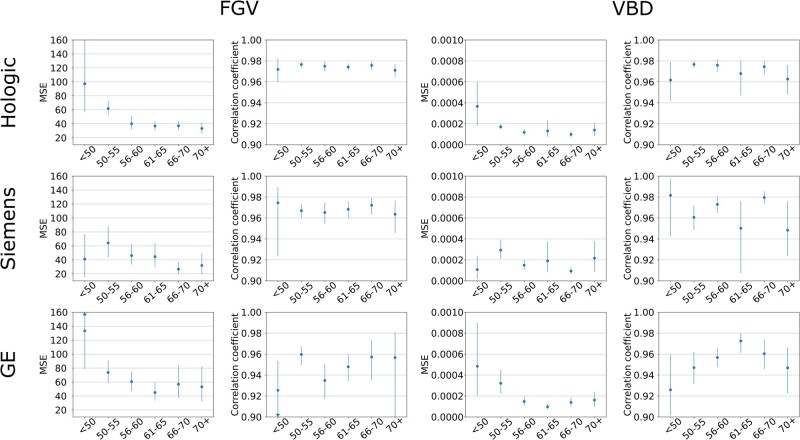
Patient-level FGV and VBD MSE and correlation coefficients, stratified by patient age at screening. Error bars show 95% CIs.

**Figure 7:**
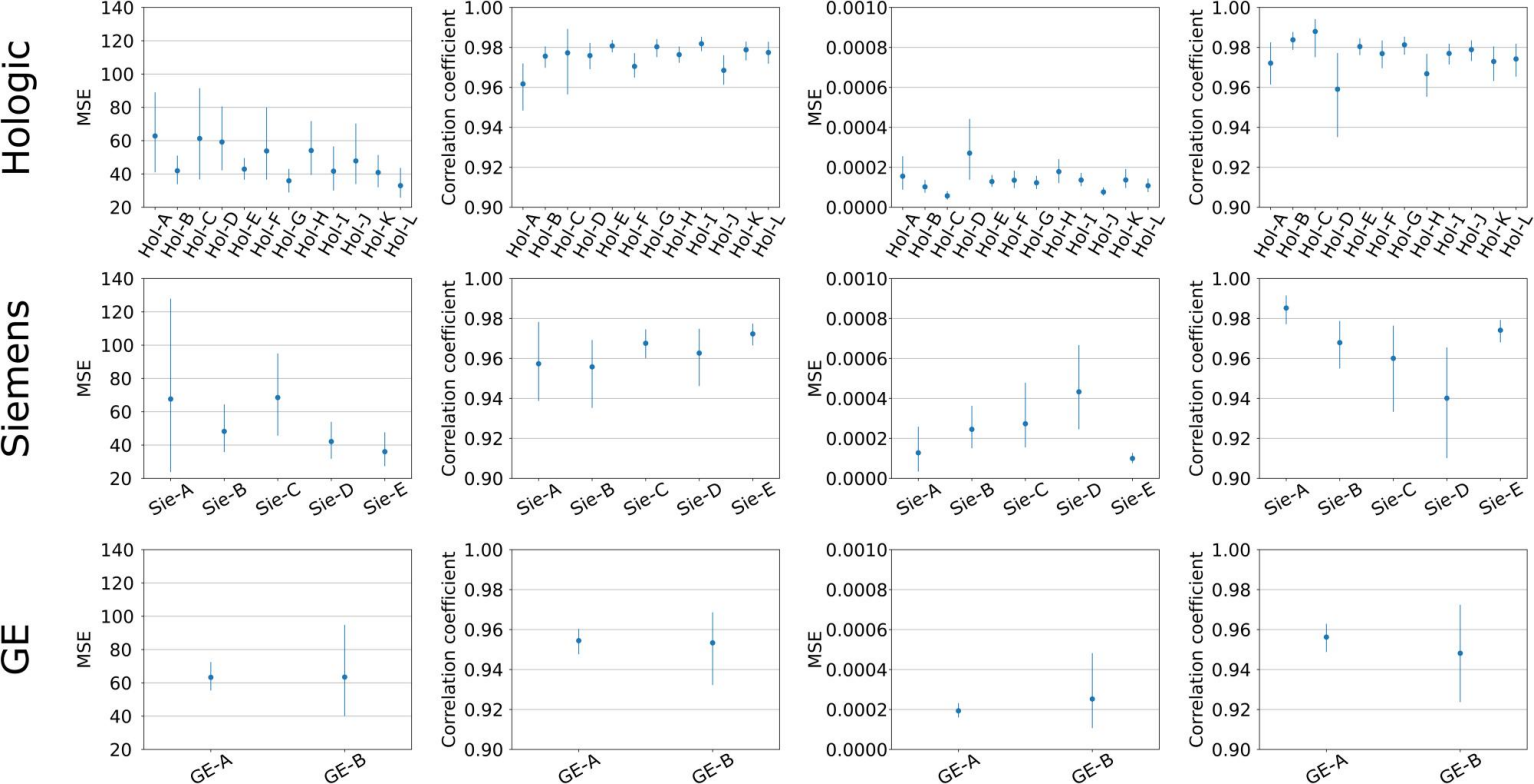
Patient-level FGV and VBD MSE and correlation coefficients, stratified by software version. Error bars show 95% CIs. Note that software versions with <50 test patients were excluded for clarity.

Figures 5-7 show the patient-level AI performance for each manufacturer broken down by screening site, patient age, and image processing software version (see Appendix SIII for a detailed description of software versions). Across screening sites MSE and correlation coefficients were generally consistent across manufacturers, with the exception of the Siemens models, where performance was consistently superior for site 4 compared to site 3. For patient age, the AI generally produced higher MSE values for younger patients. Again, correlation coefficients were typically consistent across ages, except for GE VBD values, which showed a clear peak in correlation for patients aged 61-65 years.

## Discussion

In this work, we trained and evaluated several AI models to enable the automatic quantification of FGV and VBD parameters from processed mammograms for the 3 main manufacturers of mammographic equipment in the NHS Screening Programme. Compared to previous works,^17^^,^^18^ we utilized larger training datasets and preserved useful spatial information from images by resizing to a fixed pixel size and including compressed breast thickness as a model input.

Experimental results showed consistently good performance for the BV task, which is expected given the relative ease of the task. In preliminary work (Appendix SIV), we found that BV and FGV performances improved by resizing images to a fixed pixel size and including compressed breast thickness, allowing the model to link 2D areas to 3D volumes.

We found that AI models tended to underestimate FGV for high-FGV patients, an observation that held consistently across all manufacturers. Anecdotally, we observed that the image processing for high-FGV mammograms may make dense regions appear non-dense in order to increase local contrast. While this may be useful for cancer detection performed by human observers, this processing represents a loss of information that hinders the estimation of density from processed mammograms. It should also be noted that the Volpara algorithm used to generate ground-truth values is itself imperfect, demonstrating reduced accuracy compared to MRI in high-density breasts, with an overall Volpara-to-MR correlation coefficient of 0.85.^16^ This is possibly due to the fact that Volpara relies on the identification of fat-only pixels for use as a reference in its physics model. As the breast density increases, the ability to detect such a reference pixel may degrade. Ultimately, while Volpara produces good estimates of density from unprocessed mammograms, training breast density AI tools with ground-truth values derived from MR images would be likely to produce superior models. However, to date, there are no suitably large-scale paired mammography/MR databases available, and so we believe that the presented approach using Volpara for ground-truth generation represents the current state-of-the-art for volumetric density estimation from processed mammograms.

The performance of the AI tools reported in this work is superior to previously reported studies estimating volumetric parameters from processed mammograms. For example, in the most directly comparable study in the literature, Warren et al^18^ reported patient-level Pearson correlation coefficients using Hologic images of 0.97, 0.94, and 0.91-0.94 for BV, FGV, and VBD estimation, respectively. The corresponding values produced by the presented Hologic models are 0.999, 0.976, and 0.974. This improvement can be attributed to (1) the increased dataset size and (2) careful use of physical information present in images, that is, pixel size and compressed breast thickness.

As is common in deep learning, we found that models trained with less training data performed worse overall than those with more training data. Consequently, our Siemens and particularly GE models were not as performant as the Hologic models, although they did still show high patient-level correlation coefficients >0.95 for both FGV and VBD estimation. Additional analysis (Appendix SV) showed that there was significant inter-manufacturer variation in AI performance when dataset sizes were matched, indicating that part of the performance variation between manufacturers arises from other differences in the data, for example, demographics or varying image appearance between manufacturers, eg, specific image processing pipelines or hardware specifications. For example, different image processing pipelines can variously enhance or suppress the fat/fibroglandular tissue contrast or the skin line, which can impact the AI tools’ estimation of volumetric density.

When broken down by age and screening episode type, we found that the AI tools produced higher errors in younger, first-round patients. This can be attributed to the fact that younger women tend to have denser breasts,^23^^,^^24^ which can raise error levels in 2 ways: (1) through the previously mentioned tendency of the AI models to underestimate FGV for high-FGV images, and (2) through the fact that for large ground-truth values, the same relative error equates to a larger absolute error.

For Siemens and GE models, we observed generally larger variations in performance in our subgroup analysis (across screening sites for Siemens models and across ages for GE models). For Siemens models, this may be due to variations in image processing software version between the sites, related to the year of image acquisition. Another potential cause of higher performance variability for these two manufacturers is that as the amount of data reduces, the validation dataset used for model selection becomes less representative, leading to poorer generalization on the hold-out test-set. It is difficult to definitively establish the causes of these deviations though, as there are relevant site demographic details not captured in the OPTIMAM database (eg, body mass index, deprivation). Before using the presented models in other studies, preliminary analyses should be performed to compare development and study-specific datasets in order to evaluate model applicability. Training future models with larger datasets is expected to alleviate this issue.

One potential limitation of the current work is that models were trained for each manufacturer separately. Ideally, a single cross-vendor model would be produced that performed well on each manufacturer. However, this is impractical due to (1) the large variations in the amount of data for each manufacturer, making the creation of a well-balanced training set difficult and (2) the significant variations in image appearance and processing that exist between manufacturers. Recent studies suggest that there may even be variations between manufacturers in the distribution of volumetric density obtained from unprocessed images,^25^ which would contribute further to the cross-manufacturer variability.

Overall, this work presents the development and evaluation of AI tools for the estimation of FGV and VBD from 2D mammograms. These tools consistently outperform previously published models in BV, FGV, and VBD estimation, achieving patient-level correlation coefficients >0.95 in all cases. In addition, we have calculated gradients and intercepts of all lines of best fit to allow calibration of AI-predicted volumetric values to Volpara values. This is particularly important in future risk-prediction studies that include data from multiple manufacturers, since naively applying risk stratification thresholds to raw AI outputs could produce inconsistent results. Future work will include further performance improvements as the amount of available mammographic data increases, exploring the collection and incorporation of MR-based ground-truth data, and investigating methods to produce effective cross-manufacturer tools.

Despite their limitations, we anticipate that the presented tools will open up research opportunities in breast cancer epidemiology and risk estimation. The ability to estimate FGV and VBD from processed images allows the use of retrospective clinical data in such studies, and empowers researchers without access to unprocessed mammograms to pursue similar avenues of research. The presented tools are suitable for both batch processing large datasets and on-demand/periodic processing of data streams and support local and cloud deployment. With this in mind, the developed tools will be made available to other researchers upon reasonable request, subject to research agreements being in place.

## Supplementary Material

ubag009_Supplementary_Data
