# Supplementary material for: Development and evaluation of artificial intelligence tools to estimate volumetric breast density from processed 2D mammograms
Source: BJR Artif Intell. 2026 Apr 28;3(1):ubag009. doi: 10.1093/bjrai/ubag009 (PMC13187629; doi:10.1093/bjrai/ubag009)
Supplement: ubag009_Supplementary_Data [file ubag009_supplementary_data.zip › Appendices.docx]

**Appendix I: Network Architecture**

The general network architecture utilised in this work is shown in Figure S1. This architecture was previously evaluated for volumetric density estimation^18^ and receives as input one mammographic view (repeated into 3 channels) and returns a single scalar value. This work introduces the inclusion of the compressed breast thickness as an additional model input to improve performance by providing additional spatial information. By training different instances of this architecture with different view-manufacturer-target combinations, 12 image-level models were obtained.

| 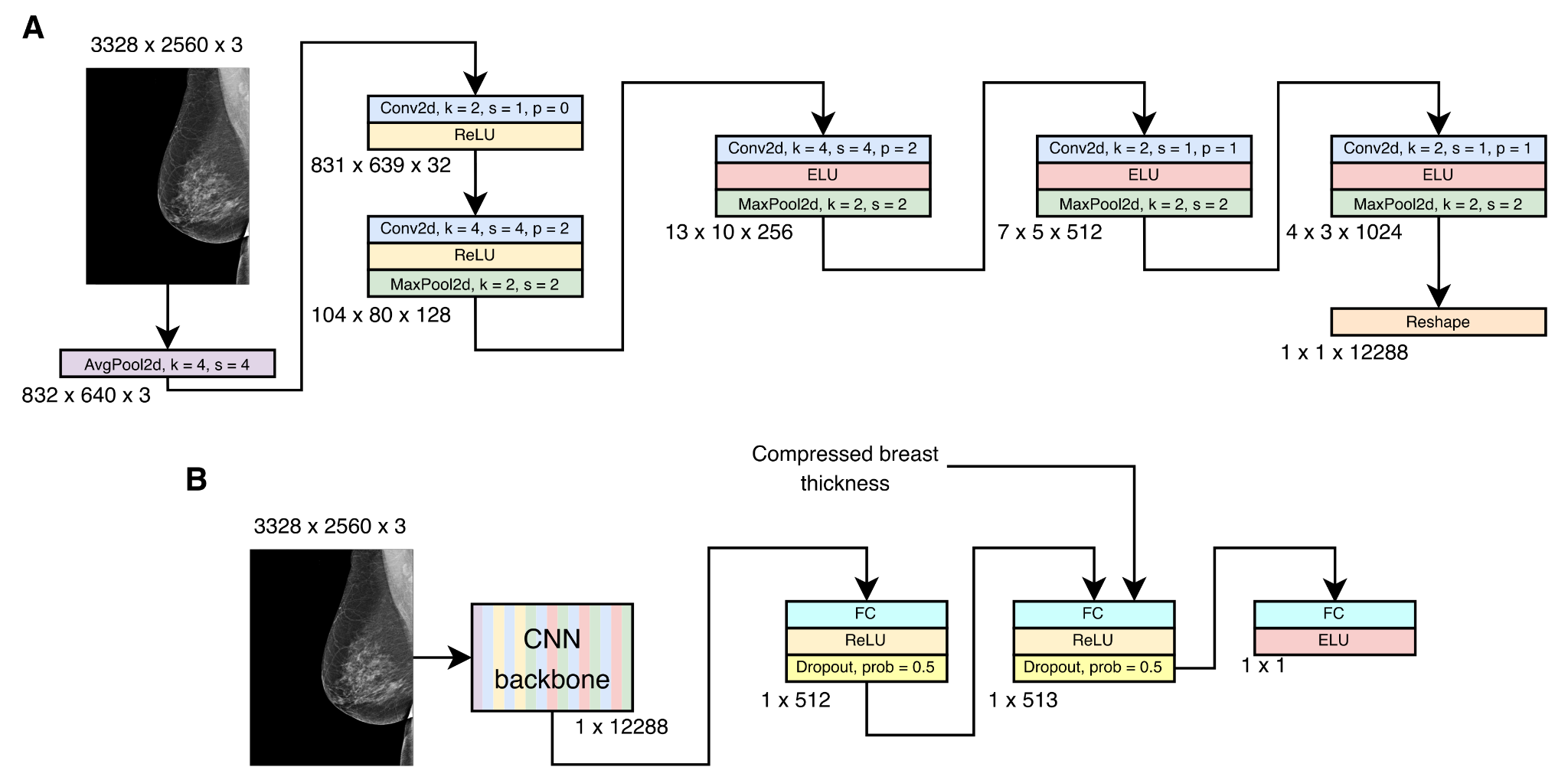 |
| --- |
| Figure S1: Network architecture used to train all volumetric density AI models. **A:** The CNN component of the model comprised a series of 2D convolutions and max pooling operations, producing a 12,288 element vector per input image. **B:** The output from the CNN is passed through a series of fully connected (FC) layers, along with the compressed breast thickness to produce a single scalar value per input image. Abbreviations: AvgPool2d = 2D average pooling, Conv2d = 2D convolution, ReLU = rectified linear unit, MaxPool2d = 2D max pooling, ELU = exponential linear unit, FC = fully connected. k = kernel size, s = stride, p = padding. Dimensions of inputs/activation maps are shown after the relevant layer in the format height x width x number of channels. |

**Appendix II: Validation MSE Curves and Epoch Selection**

| 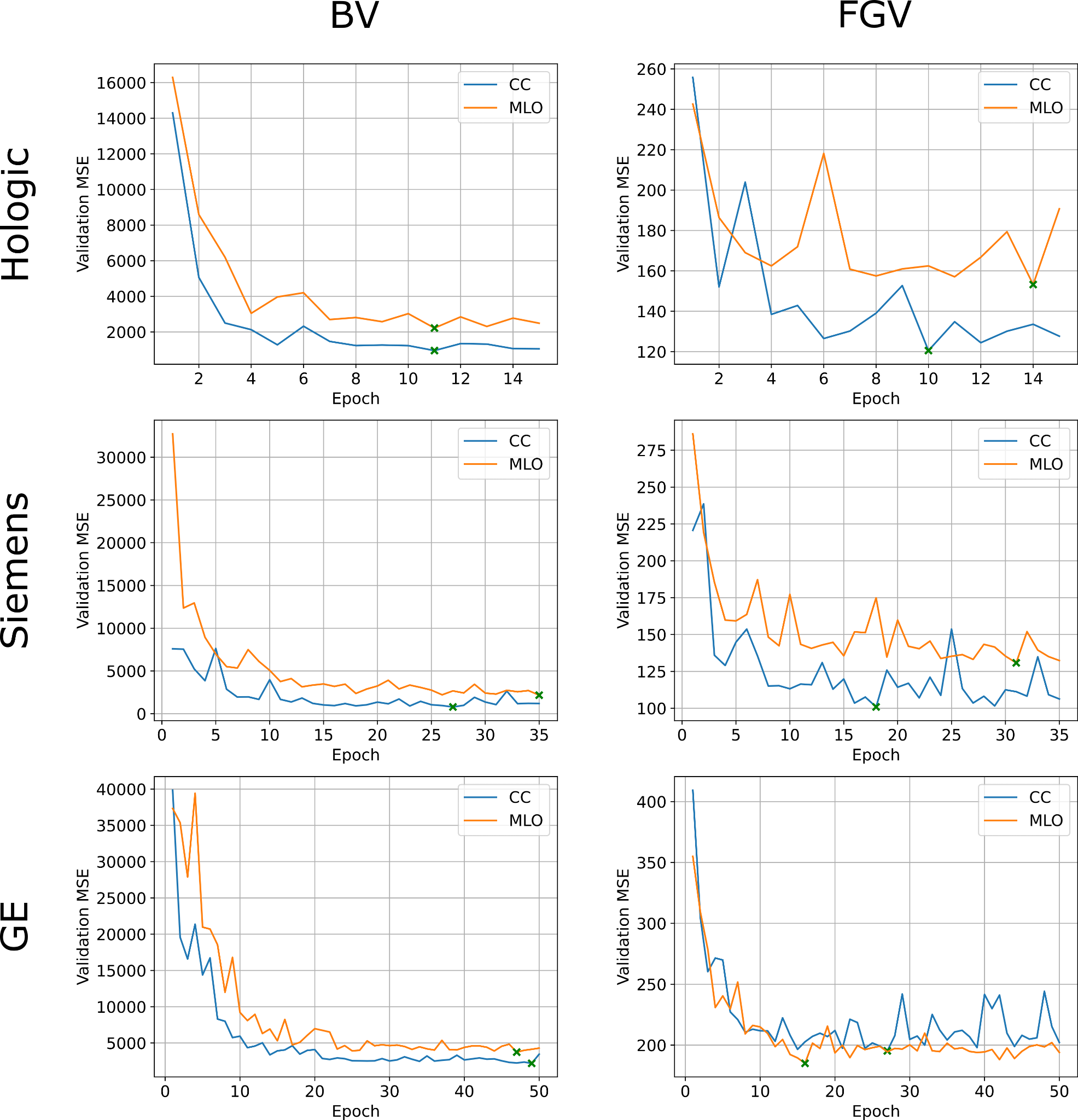 |
| --- |
| Figure S2: Validation MSE at the end of each training epoch for the presented image-level models. Crosses indicate the minimum MSE, corresponding to the final models that were evaluated on the test datasets. |

**Appendix III: Software Version Lookup**

Table S1: Lookup table mapping short software version names as used in Figure 7 to the software version name recorded in the DICOM headers. Note that for Siemens images and Hologic images with software version beginning “AWS:1.”, the software version string was truncated to increase the amount of data per software version. For other Hologic and GE images the full string was retained.

| **Short name**  **(c.f. Figure 7)** | **Full name (from DICOM header)** |
| --- | --- |
| Hol-A | AWS:1.10 |
| Hol-B | AWS:1.11 |
| Hol-C | AWS:1.3 |
| Hol-D | AWS:1.4 |
| Hol-E | AWS:1.7 |
| Hol-F | AWS:1.8 |
| Hol-G | AWS:1.9 |
| Hol-H | AWS:MAMMODROC_3_4_1_8, PXCM:1.4.0.7, ARR:1.7.3.10, IP:4.9.0 |
| Hol-I | AWS:MAMMODROC_3_4_1_8, PXCM:1.4.0.7, ARR:1.7.3.10, IP:4.9.2 |
| Hol-J | AWS:MAMMODROC_3_4_1_8, PXCM:1.4.0.7, ARR:1.7.4.7, IP:4.9.0 |
| Hol-K | AWS:MAMMODROC_3_4_1_8, PXCM:1.4.1.0, ARR:1.7.3.10, IP:4.9.2 |
| Hol-L | AWS:MAMMODROC_3_4_2_9, PXCM:1.4.1.0, ARR:1.7.4.7, IP:4.13.3 |
| Sie-A | VB30B |
| Sie-B | VB60C |
| Sie-C | VB60D |
| Sie-D | VC10E |
| Sie-E | VC20G |
| GE-A | Ads Application Package VERSION ADS_53.40 |
| GE-B | Ads Application Package VERSION ADS_55.30 |

**Appendix IV: Effect of Including Breast Thickness and Resizing to a Fixed Pixel Size**

This work introduced two major changes in how images are processed by the AI tool compared to previously reported work^18^, namely 1) the inclusion of compressed breast thickness as an additional input and 2) resizing images to a fixed pixel size prior to padding/cropping. To quantify the effect of these changes, the Hologic BV-CC and FGV-CC models reported in Figure 1 and Table 2 were re-trained with the prior method, i.e. without compressed breast thickness or a fixed resize pixel size. No other component of the training process or epoch selection was modified. Table S2 shows the results in the form of test-set MSE and correlation coefficients. The model presented in this work significantly outperformed the prior method, both in terms of MSE and correlation coefficient, for both the BV-CC and FGV-CC tasks.

Table S2: MSE and correlation coefficient values for Hologic BV-CC and FGV-CC models trained without the compressed thickness input or resizing to a fixed pixel size.

|  | **Presented models (Figure 1 and Table 2)** | | **Without compressed breast thickness or fixed resize pixel size** | |
| --- | --- | --- | --- | --- |
|  | **MSE** | **Correlation coefficient** | **MSE** | **Correlation coefficient** |
| **BV-CC** | 982 (934,1033) | 0.998 (0.998,0.998) | 9734 (9373,10120) | 0.983 (0.982,0.984) |
| **FGV-CC** | 106 (97,115) | 0.947 (0.943,0.950) | 187 (174,203) | 0.917 (0.913,0.921) |

**Appendix V: Effect of Dataset Size on Model Performance**

To investigate the effect of the dataset size on the AI model performance, the Hologic training and validation datasets were subsampled to match the number of patients available for the least prevalent manufacturer, GE. This resulted in a subsampled Hologic dataset with 4891 training and 1631 validation patients. The test set was not subsampled to improve statistical power of comparisons. Four image-level models (BV-CC, BV-MLO, FGV-CC, FGV-MLO) were trained with the same method outlined in Section II:B using the subsampled Hologic dataset, and patient-level performance evaluated on the full Hologic test dataset. Table S3 shows the patient-level correlation coefficients for models trained with the subsampled Hologic dataset, full Hologic dataset, and the GE dataset.

Table S3: Patient-level correlation coefficients for the subsampled and full Hologic models, evaluated on the same full test set. For FGV and VBD estimation, a drop in performance is evident when using the subsampled dataset. BV estimation performed similarly in both cases. GE results are also included, showing Hologic models performed better than GE models for a fixed amount of training and validation data.

|  | **Full Hologic dataset**  **(27720 train, 9246 validation)** | **Subsampled Hologic dataset**  **(4891 train, 1631 validation)** | **Full GE dataset**  **(4891 train, 1631 validation)** |
| --- | --- | --- | --- |
| **BV** | 0.999 (0.999,0.999) | 0.999 (0.999,0.999) | 0.998 (0.998,0.998) |
| **FGV** | 0.976 (0.975,0.978) | 0.964 (0.961,0.966) | 0.954 (0.947,0.959) |
| **VBD** | 0.974 (0.969,0.977) | 0.966 (0.963,0.969) | 0.954 (0.947,0.961) |
