# Supplementary figures and images for: Development and evaluation of artificial intelligence tools to estimate volumetric breast density from processed 2D mammograms
Source: BJR Artif Intell. 2026 Apr 28;3(1):ubag009. doi: 10.1093/bjrai/ubag009 (PMC13187629; doi:10.1093/bjrai/ubag009)

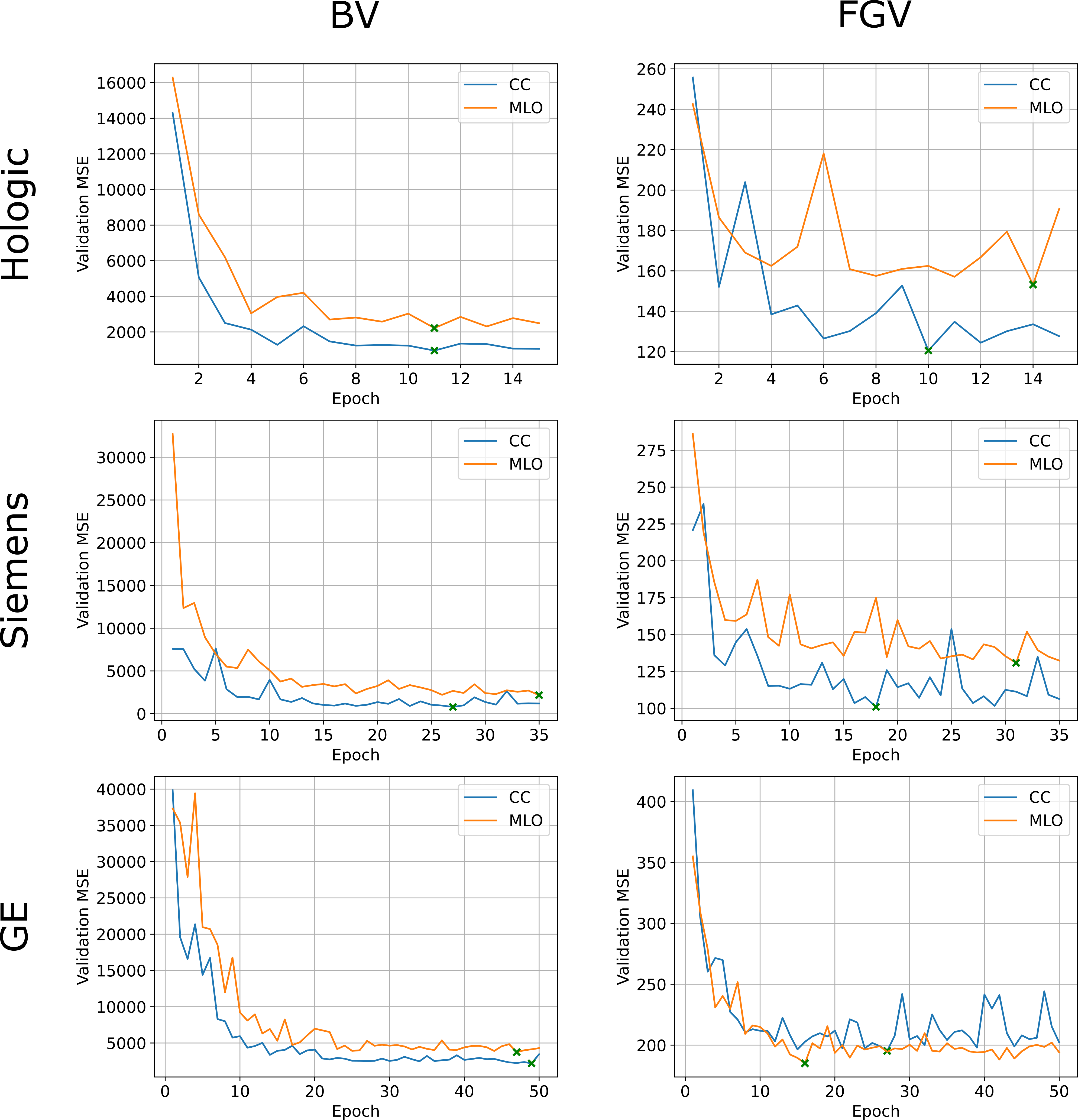

Supplement: ubag009_Supplementary_Data [file ubag009_supplementary_data.zip › fig_s2.png]

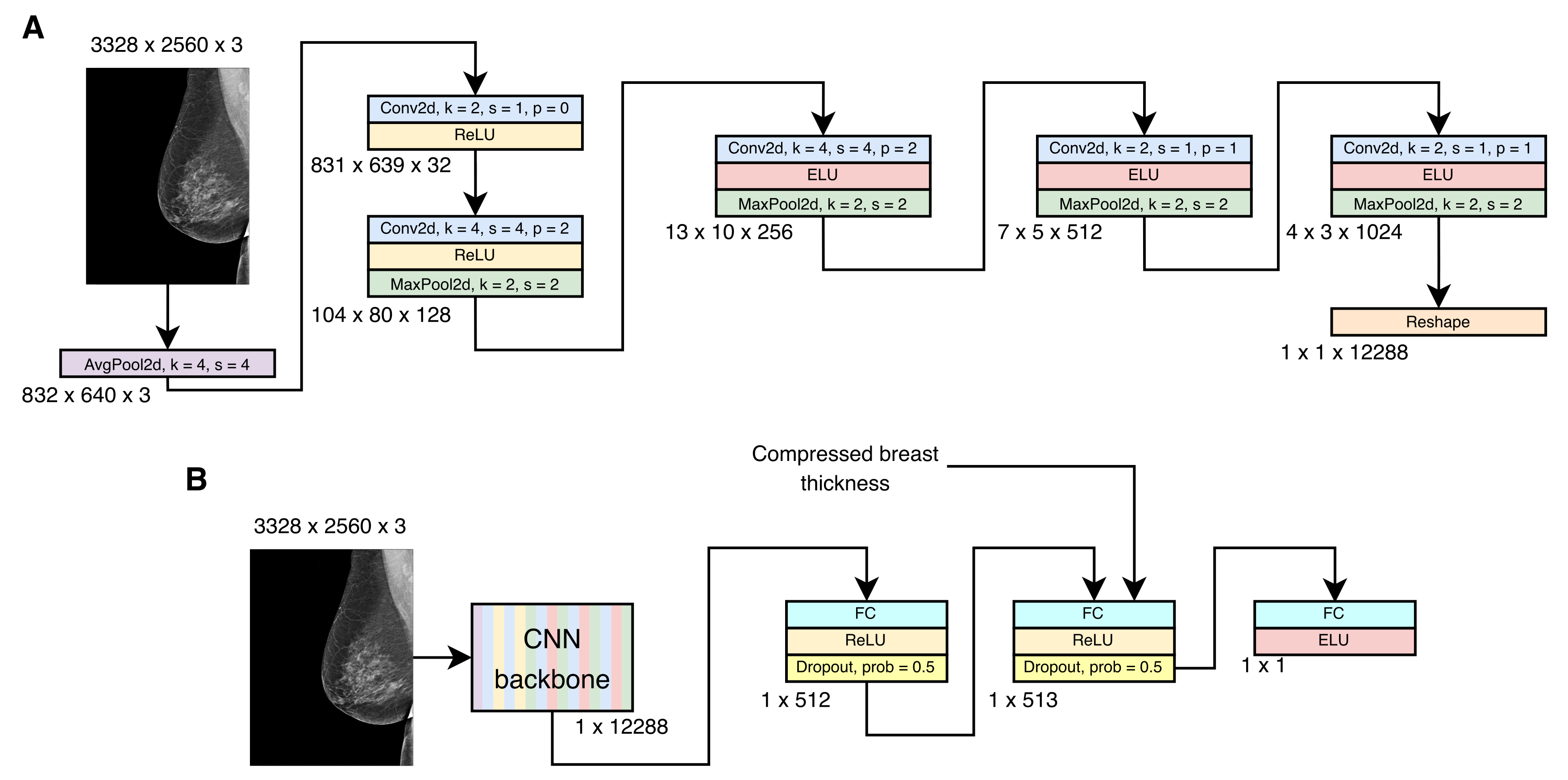

Supplement: ubag009_Supplementary_Data [file ubag009_supplementary_data.zip › fig_s1.png]
